# Familial Mediterranean fever may mimic acute appendicitis in children

**DOI:** 10.1007/s00383-022-05153-8

**Published:** 2022-06-23

**Authors:** Per Wekell, Tomas Wester

**Affiliations:** 1 Department of Pediatrics, NU-Hospital Group, Uddevalla, Sweden; 2 Department of Pediatrics, Sahlgrenska Academy, Institute of Clinical Sciences, University of Gothenburg, Gothenburg, Sweden; 3 Department of Pediatric Surgery, Karolinska University Hospital, Stockholm, Sweden; 4 Department of Women’s and Children’s Health, Karolinska Institutet, Stockholm, Sweden

**Keywords:** Familial Mediterranean fever, Appendicitis, Appendectomy, Bowel obstruction, Children

## Abstract

Acute appendicitis is the most common surgical emergency in children. Diagnosis and management are often straightforward. However, familial Mediterranean fever is an important condition to consider in the assessment of children with acute abdominal pain, particularly in children with an origin in eastern Mediterranean basin where the disease is common. The key feature of familial Mediterranean fever is relapsing episodes of fever and serositis including peritonitis, pleurisy, or arthritis. The disease is treated with colchicine that prevents acute attacks, control subclinical inflammation between the attacks and the long-term complication of amyloidosis. The acute attacks may be a challenge to identify and distinguish from other causes of acute abdomen, including acute appendicitis, but also small bowel obstruction. Ultrasound and CT scan findings are nonspecific during acute attacks of familial Mediterranean fever, but imaging is useful to identify acute appendicitis and small bowel obstruction. The purpose of this article was to increase the awareness and knowledge of familial Mediterranean fever and provide support for the paediatric surgeon in the clinical care of these children in parts of the world where familial Mediterranean fever is rare.

## Introduction

Yara is a 13-year-old girl of Turkish origin who was admitted to your ward with high fever and abdominal pain. On admission the physical examination showed sign of peritonitis but was otherwise normal. C-reactive protein (CRP) was 18.6 mg/dL, liver enzymes, urine dipstick and serum creatinine were all normal. You kept Yara under “close observation” - despite repeated physical and two ultrasound examinations - you could not exclude appendicitis. When she underwent explorative laparoscopy there was general peritonitis with a normal appendix. You realised that you had a patient with peritonitis that you couldn’t explain. To your relief, Yara recovered spontaneously during the next 24 hours. Puzzled by the situation and possible differential diagnosis - you asked if she have had similar attacks of fever and abdominal pain before? And that, changed her life….

We live in a globalised world where genetic conditions previously unheard of, or rarely seen, have become clinical realities for many of us. One such condition is familial Mediterranean fever (FMF), which is of particular importance for paediatric surgeons, as the key feature of the condition is recurrent episodes of fever and peritonitis—mimicking appendicitis. Despite this, FMF is only briefly mentioned in standard textbooks of paediatric surgery [1]. The aim of this article was to increase the awareness and knowledge of FMF among paediatrics surgeons that work in parts of the world where the condition is rare.

## Acute appendicitis

Acute appendicitis is the most common surgical emergency in children. The estimated lifetime risk is 7–8% and the peak incidence occurs in the second decade of life [2, 3] The diagnosis and management are often straightforward. Typically, children present with nausea and diffuse periumbilical pain, which migrates to the right lower quadrant. Fever is common and white blood cell count, neutrophil count and C-reactive protein indicate inflammation.

## Familial Mediterranean fever an autoinflammatory disease

Familial Mediterranean fever (FMF) is the most common monogenic autoinflammatory condition, a relatively new group of diseases characterized by abnormally increased inflammation, mediated predominantly by cells and molecules of the innate immune system, with a significant host predisposition [4, 5]. The classical monogenic autoinflammatory diseases are often labelled periodic fever syndromes and depicted by recurrent self-limiting episodes of fever and inflammation in combination with symptoms like abdominal pain, pleurisy, arthritis/joint pain, fatigue and skin rash. A combination of symptoms, including the length of the episodes form the basis for the clinical diagnosis of the different conditions.

## Clinical picture of FMF

FMF is defined by recurrent attacks of fever with a duration of 6–72 h, associated with serositis; primarily peritonitis (94%), but also arthritis (54%) and pleuritis (39%) [6]. In children, the intensity of the abdominal pain may vary, from milder attacks to fulminant peritonitis. Another clue to the diagnosis may be an erysipelas-like erythema which is considered pathognomonic for FMF and primarily localised on the extensor surfaces of the leg, over the ankle joint or dorsum of the foot.

The attacks are associated with an increase in inflammatory markers. Almost all patients (90 percent) have their first attack before the age of 20 years implying that FMF is an important childhood disease [7]. Nevertheless, there is often a substantial diagnostic delay of several years even in high prevalence areas [8]. The clinical diagnosis can be verified by genetic analysis in most but not all cases. Patients are treated with daily lifelong colchicine, which in most patients prevents attacks and control subclinical inflammation between attacks [9]. Patients resistant to treatment with colchicine or that experience severe side-effects are nowadays treated with IL-1 blockade together with colchicine.

Epidemiological studies of FMF in the 1960s—before the effectiveness of colchicine was known—illustrate the severity, the high mortality and the inconceivable suffering of the patients with the disease but also the high proportion of patients that develop amyloidosis without treatment. In a study of 470 cases from Israel in 1967, Sohar and co-workers found that 26% of the patients had developed amyloidosis, 90% before the age of 40 and 15% had died in amyloidosis [7]. In children amyloidosis is rare but it is important to be aware of that amyloidosis starts in the kidneys and that proteinuria is an early sign. Today, amyloidosis is prevented in almost all patients that are treated with colchicine [9].

## Genetics of FMF

The gene that cause FMF was identified in 1997 on chromosome 16 and labelled; “MEditerranean FeVer gene” (*MEFV*) coding for the protein pyrin [10, 11]. Today over 300 variants in *MEFV* have been identified with more than 100 disease causing variants, although the four that was identified in the first studies (M680I, p.M694V, V726A and M694I) still represent the majority of clinical cases. Information on specific *MEFV* variants are available at INFEVERS online database (https://infevers.umai-montpellier.fr/web/search.php?n=1).

FMF is usually inherited as an autosomal recessive condition linked to variants in *MEFV*. The pattern of inheritance makes a negative family history common in the first child in whom FMF is diagnosed, while siblings are at a 25% risk of inheriting the condition. Positive family history is more common among those individuals who are from areas with very high prevalence and in children of consanguineous parents. In clinical practice, all patients with clinical FMF don’t have two disease causing variants. In fact, only ¾^th^ of the patients are homozygote or compound heterozygote for a genetic variant in *MEFV* and approximately 1/4^th^ have one identifiable variant or no mutation. If the latter group of patients without any variant in *MEFV* should be regarded as having FMF or not, is an issue of debate.

## Epidemiology of FMF

FMF has been reported in many populations today, but it is much more common in individuals with an eastern Mediterranean origin, mainly Jews, Armenians, Arabs and Turks. In these populations, the prevalence is often as high as 100–250 per 100,000 with an extremely high carrier frequency of approximately 1 in 5 [6]. The high prevalence will be relevant for individuals with an origin in such areas when they move to or live-in other parts of the world [12]. The total number of individuals with FMF are unknown, but rough estimates suggest that over 100,000 people suffers from the condition in the world [6, 13, 14].

## Survival advantage of MEFV carrier drives natural selection

The only plausible explanation for the high carrier^1^ rate of *MEFV* variants with many different mutations in the eastern Mediterranean basin, is that the carrier have had an evolutionary survival advantage [15]. Gram-negative toxin-producing bacteria has been proposed to contribute to the natural selection of *MEFV* variants including Clostridium difficile, Vibrio parahaemolyticus and Clostridium botulinum [16]. Recently, a study from Turkey showed that pathogenic variants in *MEFV* has a heightened resistance to infections with Yersinia pestis and that *MEFV* variants have been positively selected for in the eastern Mediterranean basin [17]. The explanation for the geographic constraint appears to be that *MEFV* variants were present in the population at the same time as episodic plague tormented the region, which was not the case globally [17].

## The disease mechanism clarifies clinical attacks and survival advantage of carrier

The mechanistic explanation for the recurrent attacks and the survival advantage has also been uncovered more than 20 years after the gene for FMF was identified. It has been shown that pyrin forms an inflammasome, which leads to activation of caspase-1 that in turn cleave pro-IL-1β (pro-IL-18) to IL-1β (IL-18) [16, 17]. Furthermore, pyrin in patients with FMF interacts less eagerly than wild-type human pyrin with a virulence factors of Yersinia pestis and thereby reduce the suppression of IL-1β production i.e., leading to increased production of IL-1β [17]. This is supported by molecular studies of leukocytes that harbouring disease-causing combinations of variants of *MEFV* and heterozygote variants from asymptomatic individuals (carrier), showing that both groups produce more IL-1 in contact with Yersinia pestis than healthy controls.

## Appendicitis and appendectomy in patients with FMF

The difficult task to differentiate FMF attacks from acute appendicitis is illustrated by that FMF patients were previously offered elective laparoscopic appendectomy [18]. The rational was to avoid that future appendicitis would be assigned to FMF attacks, avoid delayed diagnosis of appendicitis and prevent perforation. The strategy has been abandoned today, mainly due to the risk that the surgery may provoke an attack of FMF and contribute to the development of abdominal adhesions in the future, considering that patients with FMF already are predisposed to peritoneal inflammation [7, 19].

Nevertheless, even when elective appendectomy is disregarded, appendectomy is still common in patients with FMF. One retrospective study from Israel by Lidar et al., reported appendectomy in 39% of 182 patients with FMF, which was considered roughly twice as common compared to the general population in the area (12–25%) [20]. Approximately 70% of these patients were operated before the diagnosis of FMF was made, that is, 27% of FMF patients underwent appendectomy before they received their diagnosis of FMF. In retrospective studies from Turkey, the frequency of appendectomy prior to diagnosis of FMF was lower in one and on the same level in two studies, that is 11% of 159, 26% of 197 and 27% of 254 patients with FMF [21–23]. In both Israel and Turkey genetic variants linked to severe disease were more common in patients that had undergone appendectomy [20–22]. Furthermore, patients that have undergone appendectomy require higher colchicine doses, had a lower rate of response to colchicine and greater need for IL-1 blockade [24].

In the above cohort from Israel by Lidar et al., 41% of the patients with FMF had a normal appendix at appendectomy, 18% had an inflamed appendix, and 8% had peri-appendicitis (fourfold higher than in the general population) [20]. In the paper, the author hypothesised that periappendicitis is pathognomonic for FMF, but this needs to be further studied. In the cohort, 6% of the patients had undergone elective appendectomy and data were missing in 26% of the patients.

The high rate of a normal appendix after appendectomy in patients with FMF prompted researchers from Ankara, Turkey to investigate, how common FMF was in patient with normal appendix after appendectomy. They studied 278 patients who had surgery for suspected appendicitis out of which 28 patients had no evidence of acute appendicitis. Out of these 28 patients, two (7.7%) were diagnosed with FMF [25]. The numbers are small but the result is important as it indicates that FMF should be considered in patients with normal appendix after appendectomy, particularly in patients with an origin in high prevalence areas.

The incidence rate of negative appendectomy in patients with FMF is difficult to interpret. Traditionally, the incidence rate of negative appendectomy was 20% to 30%, without selection for FMF [26]. After introduction of imaging, the incidence rate of negative appendectomies has dropped significantly. A recent review showed that the current overall incidence rate of negative appendectomy in children was 0–6.2% [27]. Hence, the incidence rate of negative appendectomy in patients with FMF needs to be further evaluated in clinical settings where imaging is part of routine care.

## Small bowel obstruction in patients with FMF

In addition to acute appendicitis, there has been a concern that an increased risk for abdominal adhesions in patients with FMF lead to small bowel obstruction and strangulation. Small bowel obstruction due to adhesions are more common in patients with FMF than in individuals without FMF. The risk in 355 children with FMF without prior laparotomy was retrospectively estimated to 3% with no mortality during a mean follow-up period of 8 years [28]. In contrast to an abdominal attack of FMF, small bowel adhesive obstruction is a progressive clinical condition, which is important to diagnose and manage appropriately to prevent deterioration.

## Diagnostic workup and differential of an abdominal attack of FMF in the emergency room

An abdominal attack of FMF may start with diffuse pain that disseminate over the whole abdomen. In severe attacks, your physical examination will reveal signs of peritonitis (rigidity), decreased peristalsis and distention of the abdomen. Sometimes peritonitis is localized, which adds to your differential diagnosis. The inflammatory reaction will often lead to constipation, but occasionally the patient will have diarrhoea [7, 29].

To make the distinction between an abdominal attack of FMF and appendicitis—meticulous observation of the clinical progression is vital. In a typical FMF attack, signs and symptoms reach its maximum within a few hours, stabilize for 12–24 h and then gradually diminish over 6–12 h, to completely disappear within the next 24–72 h [7]. Abdominal imaging adds to the diagnostic process, most importantly by ruling out other causes (see below).

Older children with an established FMF diagnoses may be able to recognise the normal development of an attack and will often reveal when the disease progression deviates from what they are used to. The key to identifying children with FMF are to ask if the child has had similar attacks before, including attacks associated with pleurisy or arthritis.

## Laboratory investigation during an attack

During an attack of FMF the inflammatory markers are increased, this includes C-reactive protein (CRP), serum amyloid-A (SAA), leukocytes and neutrophilic granulocytes. SAA is a sensitive marker of inflammation in autoinflammatory diseases and produced in the liver in response to the secretion of proinflammatory cytokines. SAA is not specific to autoinflammation and is often elevated in other inflammatory and infectious diseases. During an attack of FMF SAA is very high—often several hundred mg /l, the level depending on the upper limit of detection in the laboratory. In a disease attack with a clear increase in CRP, analyses of SAA don’t add any essential information. Analysis of SAA can, however, be important in individuals without a substantial increase in CRP during the attacks.

Subclinical inflammation is common outside attacks of FMF. SAA is a sensitive marker of this subclinical inflammation. The protein is a precursor of amyloid A that aggregated in AA amyloidosis that first occur in the kidney. To normalise such inflammation is one of the treatment goals of FMF with the long-term aim of preventing amyloidosis [9].

## Imaging in the assessment of an abdominal FMF attack

The most important role of imagining in clinical evaluation of a suspected abdominal attack of FMF is to exclude complications or other causes, in particular appendicitis and small bowel obstruction [30]. The imaging findings of patients with abdominal attacks of FMF are nonspecific [31]. This includes plain abdominal films that frequently show small and large bowel dilation, and fluid levels. A review of abdominal ultrasound revealed that the attacks are characterized by; paralytic ileus, ascites, increased echogenicity of the mesentery and/or retroperitoneum, lymphadenopathy, hepatosplenomegaly, and bowel wall thickening [32]. CT scan is more informative than plain abdominal films and ultrasound. This is illustrated by a cohort of 14 patients with abdominal attacks that showed; engorged mesenteric vessels and/or thickened mesenteric folds in 12 patients, mesenteric lymphadenopathy in 6 and ascites in 6. In four of the latter, there was signs of local peritonitis. Other findings were splenomegaly in 3 patients, dilated small bowel loops in 2 and mural thickening av ascending colon in one [30]. Despite the non-specific findings of these imaging-studies it is important for the radiologist and the clinician to know what CT findings they could expect during an attack of FMF and to bear in mind that the rational for performing imaging is to rule out abdominal emergencies.

## When laparoscopy or laparotomy is the only way to rule out an abdominal emergency

Despite meticulous clinical observation and imaging studies explorative laparoscopy is sometimes unavoidable to exclude an abdominal emergency. If the patient suffers from an attack of FMF, your examination may expose a normal abdominal cavity or there will be prominent signs of peritonitis with oedematose and hyperaemic peritoneal folds or greater omentum. Peritoneal exudate will be sterile with a high concentration of polymorphonuclear cells and fibrin [7, 33]. There may be abdominal adhesions because of repeated episodes of abdominal inflammation [7]. Microscopic investigation of peritoneum will show vascular dilatation, oedema and polymorphonuclear leukocytes and few mononuclear cells [7].

## Treatment of an abdominal attack of FMF

Abdominal attacks of FMF are treated with cox-inhibitors such as diclofenac. Steroids have limited effects and colchicine has no place in the treatment of an attack. On demand treatment of acute attacks with IL-1 blockade during a prodrome phase may be a treatment alternative for selected cases in the future.

## Conclusion

We hope that this article can support the clinician in the assessment of children with acute abdominal pain and in the differential diagnostic process. In addition, we want to emphasise that the clinical scenario of “a child with acute abdominal pain and fever” is a golden opportunity to identify the child with suspected FMF. That is, especially in children from eastern Mediterranean basin—review if the child has a family history of FMF or a disease history of similar attacks of fever with abdominal pain, pleurisy, or arthritis.

## Footnotes

1 Symptom-free individuals with one identifiable pathogenic *MEFV* gene variant are called carriers.
